# Proteomic Analysis of the Maternal Preoptic Area in Rats

**DOI:** 10.1007/s11064-019-02755-y

**Published:** 2019-03-07

**Authors:** Edina Brigitta Udvari, Katalin Völgyi, Katalin Adrienna Kékesi, Dorina Simon, Éva Hunyadi-Gulyás, Arpád Dobolyi

**Affiliations:** 1 MTA-ELTE Laboratory of Molecular and Systems Neurobiology, Department of Physiology and Neurobiology, Hungarian Academy of Sciences and Eötvös Loránd University and Eötvös Loránd University, Budapest, Pázmány Péter sétány 1/C, 1117 Hungary; 2 Laboratory of Proteomics, Institute of Biology, Eötvös Loránd University, Budapest, Pázmány Péter sétány 1/C, 1117 Hungary; 3 Department of Anatomy, Histology and Embryology, Semmelweis University, Budapest, Tuzolto u. 58, 1094 Hungary; 4 Laboratory of Proteomics Research, Biological Research Center, Szeged, Temesvári körút 62, 6726 Hungary

**Keywords:** Maternal behavior, Depression, 2-D DIGE, Alpha-crystallin B chain—Cryab, Hormone receptor

## Abstract

The behavior of female rats changes profoundly as they become mothers. The brain region that plays a central role in this regulation is the preoptic area, and lesions in this area eliminates maternal behaviors in rodents. The molecular background of the behavioral changes has not been established yet; therefore, in the present study, we applied proteomics to compare protein level changes associated with maternal care in the rat preoptic area. Using 2-dimensional fluorescence gel electrophoresis followed by identification of altered spots with mass spectrometry, 12 proteins were found to be significantly increased, and 6 proteins showed a significantly reduced level in mothers. These results show some similarities with a previous proteomics study of the maternal medial prefrontal cortex and genomics approaches applied to the preoptic area. Gene ontological analysis suggested that most altered proteins are involved in glucose metabolism and neuroplasticity. These proteins may support the maintenance of increased neuronal activity in the preoptic area, and morphological changes in preoptic neuronal circuits are known to take place in mothers. An increase in the level of *alpha-crystallin B chain* (Cryab) was confirmed by Western blotting. This small heat shock protein may also contribute to maintaining the increased activity of preoptic neurons by stabilizing protein structures. Common regulator and target analysis of the altered proteins suggested a role of prolactin in the molecular changes in the preoptic area. These results first identified the protein level changes in the maternal preoptic area. The altered proteins contribute to the maintenance of maternal behaviors and may also be relevant to *postpartum* depression, which can occur as a molecular level maladaptation to motherhood.

## Introduction

An altered physiological state is characteristic of mothers in mammals, which is required for the support of the young offspring. In the *postpartum* period, mother rats take care of the pups; they lactate, respond to the pups, retrieve them to the nest and nurse them, and they protect them against intruders by a process called maternal aggression [1, 2]. Rat mothers also have emotional alterations during the postpartum period. They show reduced anxiety and antidepressant-like behaviors in situations unrelated to the pups and demonstrate hyporesponsiveness to stressors in their hypothalamo-pituitary-adrenal axis [1, 2]. Virgin females do not lactate and do not show affiliation towards pups or maternal behaviors [3]. The profound physiological, endocrine, and emotional changes in the maternal brain have led us to suggest an altered protein pattern in relevant brain areas. While lactation is controlled by prolactin and oxytocin release that is regulated by neurons located in the hypothalamic arcuate and paraventricular/supraoptic nuclei, respectively [4], the behavioral control is exerted by a more rostral region of the hypothalamus, the preoptic area (POA) [5, 6], and affective changes may also involve the medial prefrontal cortex [7, 8]. In a previous study, we compared the maternal and nonmaternal proteome in the medial prefrontal cortex, and we identified several altered proteins [9]. However, other maternal brain regions have not been investigated yet by using proteomics.

A large body of evidence suggests that maternal behaviors are regulated by neurons located in the POA. Lesions in this part of the hypothalamus result in the cessation of all maternal behaviors [10, 11]. In turn, different parts of the region, including the medial preoptic nucleus, dorsolaterally located parts of the medial POA as well as the ventral subdivision of the bed nucleus of the stria terminalis containing neurons, are activated in mother rats in response to pup exposure [12, 13]. Since c-fos-positive cells are always present in the lactating rats, it can be assumed that these POA neurons are constitutively active in the normal *postpartum* period [14]. While c-fos activation of preoptic neurons is likely the result of direct neuronal input evoked by the pups and is responsible for the maintenance of maternal behaviors [15–19], the area is also rich in hormone receptors, such as estrogen and prolactin receptors, known to initiate maternal motivation in the perinatal period [20–23].

While the involvement of the POA in orchestrating maternal behavior is well-established, little is known about the underlying molecular mechanisms except that the maternal alterations are not simply due to hormone level changes [24–26]. Therefore, we hypothesized that the behavioral changes in mothers are accompanied by alterations of the brain at the protein level. To focus on protein level changes associated with maternal care and to eliminate pregnancy-induced alterations, the control group consisted of mother rats whose litter was removed immediately after delivery. While previous studies addressed mRNA level changes in the POA [26, 27], we assumed that protein level changes may be more directly related to maternal functions and compared protein levels in brain tissue homogenates of maternal and nonmaternal rat POAs. The rats were sacrificed on the 11th *postpartum* day because the maternal behavior is abolished by this time in the pup-deprived control group [28]. Since proteomics has not been applied to reveal protein level alterations in the maternal POA, we now applied a two-dimensional differential gel electrophoresis (2-D DIGE) technique, a gel-based proteomic method, which allows for simultaneous visualization of a large number of proteins in a single gel [29, 30]. For proteomic dataset analysis, bioinformatics software and web-based tools were used [31, 32]. The altered proteins are involved in a variety of biological processes. The proteins of these processes can either regulate the altered proteins, based on information in the existing literature, or can be influenced by them. To establish these connections, we used the Pathway Studio Platform, Elsevier. This is a software frequently used for the purpose to determine the common regulators of several altered proteins and/or to identify the common targets of the altered proteins [33–36].

## Methods

### Animals

Animals were kept under standard laboratory conditions with 12 h light and 12 h dark periods (the light was on from 08.00 a.m. to 08.00 p.m.). Food and water were supplied *ad libitum*. The care and experimentation of all animals conformed to the Hungarian Act of Animal Care and Experimentation (1998, XXVIII) and to the guidelines of the European Communities Council Directive, 86/609/EEC. The approval number of the Research Ethics Committee is PE/EA/117-8/2018.

A total of 24 Wistar rats were used in the study. The number of pups from rat dams was adjusted to 8 pups for the mothers rearing their litter. In the control group (pup-deprived animals), the pups were removed from the mother rats immediately after parturition. For perfusions and dissections, rats were anesthetized with an intramuscular injection of anesthetic mix containing 0.2 ml/300 g body weight ketamine (100 mg/ml) and 0.2 ml/300 g body weight xylazine (20 mg/ml) and sacrificed on the 11th *postpartum* day. From the freshly removed brains, the POA region was immediately dissected and used for proteomics and Western blot (WB) analysis.

### Microdissection of Brain Tissue Samples

Brains of 12 mothers with litters and 12 pup-deprived control rats were dissected. Then, 2 mm thick coronal brain sections were prepared with razor blade cuts. The anterior level of the optic chiasm (bregma level: + 0.3 mm) was used to determine the anterior level of the cut. This white matter tract allows for a very precise identification of the antero-posterior level. Thus, the POA was dissected from the section between bregma levels + 0.3 to − 1.7 mm. From this 2 mm thick coronal brain section, a horizontal cut immediately above the anterior commissure (a large white matter tract allowing for very precise identification) removed the area above the POA. Then, 2 sagittal cuts were applied 2 mm lateral to the midline on both sides of the brain to finally dissect tissue blocks that contained the POA In addition to the POA, the tissue block also contained small parts of adjacent brain structures including parts of the diagonal band of Broca, the anterior commissure, the optic chiasm, the ventral pallidum, and the anterior hypothalamus. These structures were included because selectively dissecting the POA would have resulted in less precise borders of the block potentially compromising the replicability of the dissection. In addition, the brain areas immediately adjacent to the POA were not expected to change in relation to maternal activity based on their established functions. The dissected tissue samples were quickly frozen on dry ice and stored at − 80 °C until the analysis.

### Proteomic Analysis by Two-Dimensional Differential Gel Electrophoresis (2-D DIGE)

For proteomics, 6 mothers with litters and 6 pup-deprived control rats were used, while the other 12 tissue blocks were saved for Western blot validation. The 2-D DIGE and the mass spectrometry analysis were performed similarly to our previous study on medial prefrontal cortical (mPFC) samples [9]. The 2-D DIGE Minimal Dye labeling method was used. Equipment and software were supplied by GE Healthcare, Little Chalfont, UK. Briefly, proteins of the homogenized brain tissues were acetone-precipitated, and then resuspended in a lysis buffer containing 7 M urea, 2 M thiourea, 4% CHAPS, 20 mM Tris and 5 mM magnesium-acetate. The pH of the samples was adjusted to 8.5 and the protein concentrations were determined with a 2-D Quant Kit. Samples of 50 µg were labeled using a CyDye DIGE Fluor Minimal Labeling Kit according to the manufacturer’s instructions. The maternal and pup-deprived tissue samples were labeled with Cy3 and Cy5, respectively, and the internal standard was labeled with Cy2 fluorescent dye. The three differently labeled protein samples were merged and six mixtures (six gels simultaneously) were run. Isoelectric focusing was performed on 24 cm IPG strips (pH 3–10 NL) for 24 h in an Ettan IPGphor instrument to attain a total of 80 kVh. The applied voltages were: 30 V for 3 h step, 500 V for 5 h gradient, 1000 V for 6 h gradient, 8000 V for 3 h gradient, and 8000 V for 6 h step mode. The focused proteins were reduced then alkylated in equilibrating buffer containing mercaptoethanol and iodoacetamide, respectively, for 20–20 min. Subsequently, the IPG strips were loaded onto 10% polyacrylamide gels (24 × 20 cm) and SDS–PAGE was performed using an Ettan DALT Six System. Gels were scanned with a Typhoon TRIO + scanner after selecting the appropriate lasers and filters. Gel images were visualized by ImageQuant TL software. Differential protein analysis was performed using DeCyder 2-D 7.0 software in the Differential In-gel Analysis (DIA) and Biological Variance Analysis (BVA) modules. The internal standard contained equal amounts from the same sample in all gels and fluorescence intensity changes of protein spots were normalized to the values of the corresponding internal standard. For the statistical analysis, independent Student’s *t*-test was performed to determine the significance of the protein abundance changes with a cut-off of *p* < 0.04 for each protein spot.

### Preparative Two-Dimensional Gel Electrophoresis for Protein Identification

For the identification of proteins in spots of interest, a separate preparative two-dimensional gel electrophoresis was performed using a total of 800 µg proteins per gel. Resolved protein spots were visualized by Colloidal Coomassie Blue G-250 (Merck, Darmstadt, Germany).

### Protein Identification by Mass Spectrometry

The separated protein spots were cut out from the gel and in-gel digested for mass spectrometry-based protein identification by using the protocol available on-line (http://msf.ucsf.edu/protocols.html). After reduction with dithiotreitol (DTT) and alkylation with iodoacetamide, the proteins were digested with trypsin (sequencing grade modified trypsin from pig pancreas, Promega). Tryptic peptides were subjected to LC-MS/MS analysis on an LCQ-Fleet iontrap mass spectrometer (Thermo) on-line coupled with a nano-Acquity HPLC system (Waters). Five µl out of the 10 µl peptide extracts were injected to the nano-HPLC using a trap column (Symmetry C18, 0.18 × 20 mm, 5 µm, Waters) and analyzed on a BEH300C18 1.7 µm (0.1 × 100 mm) nanoAcquity UPLC Column (Waters) using a gradient elution (10–40% of B over 30 min; B: 0.1% formic acid in acetonitrile; A: 0.1% formic acid in water). MS data were acquired in a data dependent fashion using the triple play method, and each survey scan was followed by zoom scans and CID scans (normalized collision energy: 35) of the 3 most abundant multiply charged precursor ions. The dynamic exclusion was set to 30 s. Mascot Distiller (ver: 2.2.1.0) was used to generate the MS/MS peak list files (mgf) from the raw data, and the ProteinProspector (v. 5.3.0.) search engine was used for the database search. We set the following parameters for the search: database: UniProtKB HUMAN RODENT (218,476/14,423,061 entries searched); enzyme: trypsin with a maximum 2 missed cleavage sites; fixed modifications: carbamidomethyl (C); variable modifications: acetyl (protein N-term), Gln->pyro-Glu (N-term Q), oxidation (M); peptide mass tolerance: ± 0.6 Da; fragment mass tolerance: ± 1 Da. Proteins, identified with at least 2 unique peptides (minimum peptide score: 15), were considered as a valid hit.

### Functional Clustering

Significantly altered proteins were clustered on the basis of the protein databases, such as UniProt (http://www.uniprot.org/) and Gene Ontology (http://geneontology.org/) databases. The most relevant functions of the proteins were considered for clustering.

### The Significantly Changed Protein Validation with Western Blots (WB)

For WB, 6 mothers with litters and 6 pup-deprived control rats were used. The alpha-crystallin B chain (Cryab) protein showed the highest fold change among the altered proteins in the POA. Therefore, we selected this protein for validation by Western blot. Proteins were separated with Tricine-SDS-polyacrylamide gel electrophoresis using 4% stacking and 15% resolving polyacrylamide gels then transferred to Hybond-LFP PVDF transfer membranes (GE Healthcare). The membranes were blocked with 5% BSA in Tris-buffered saline, 0.1% Tween 20 (TBS-T). The blots were incubated with goat anti-Cryab primary antibody (sc-22,391, Santa Cruz Biotechnology) at 1: 1000 dilution. Subsequently, the membranes were washed for 4 × 5 min in TBS-T followed by incubation with ECL Plex CyDye conjugated anti-goat IgG secondary antibody at 1: 2500 dilution (GE Healthcare). After the washing steps, the bands were visualized using a Typhoon TRIO + scanner. The ImageQuant TL software was used for the quantification of the fluorescence intensities. The densitometry data of protein band intensities were analyzed with ImageJ software (NIH, Bethesda). Every protein band intensity was normalized to the average band intensity of the pup-deprived control group. Differences between the two samples were statistically analyzed using independent Student’s *t*-tests.

### Bioinformatical Analysis of Significant Protein Changes

The interactions between significantly changed preoptic proteins were analyzed with Elsevier Pathway Studio Platform software. Common regulator and common target analyses were carried out for preoptic proteins to discover potential relationships based on published articles. The aim of this analysis is to find connections between our proteins and the well-known hormone receptor’s signaling pathways. We selected common regulator and target proteins having a minimum of 3 relationships with significantly changed maternal proteins from the experimental results. In the common regulator analysis, we applied the following relation filters: direct regulation, expression, promoter binding, quantitative change. In the common target analyses the following relation filters were used: direct regulation, expression, promoter binding and quantitative change.

## Results

### Proteomic Identification of Maternally Altered Proteins

**Fig. 1:**
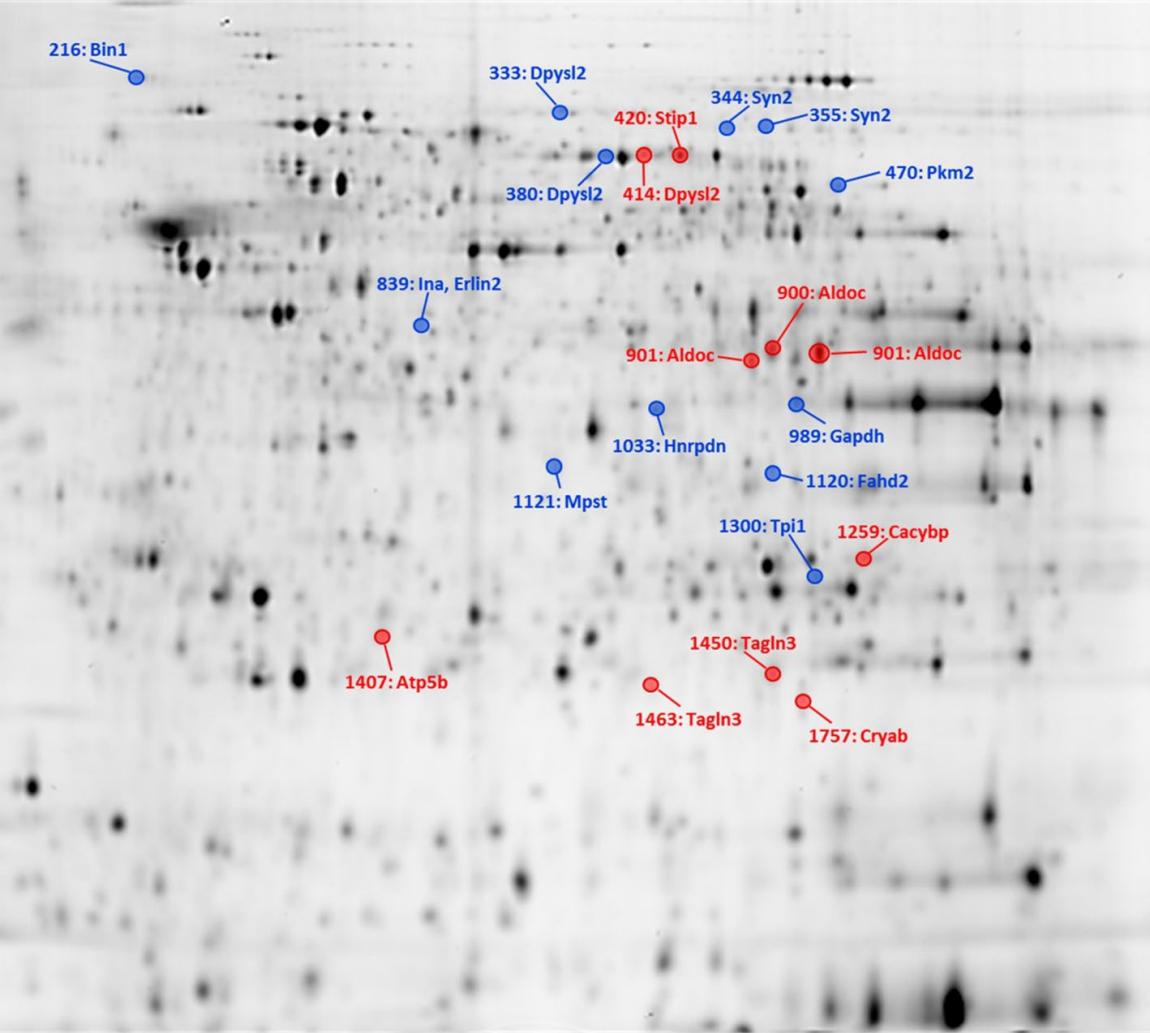
Representative 2-D DIGE gel with labeled locations of significantly altered hypothalamic preoptic area protein spots. The spot number and the major identified genes in the particular spot are shown. Red and blue circles indicate significantly increased and decreased protein spot changes of dams, respectively. (Color figure online)

Approximately 1200 spots were separated on the gel from rat brain preoptic samples, among which 400 were quantitatively measurable between mother and pup-deprived rats. A total of 24 of them exhibited significant changes between the groups. Proteins of significantly changed spots were identified with LC-MS/MS resulting in a total of 18 proteins with maternally altered levels in the POA region (Fig. 6). Some of the proteins were present in more than one spot, suggesting their posttranslational modification or the presence of protein isoforms. Thus, the identity of only 18 different altered proteins was successfully determined with MS. Among these 18 spots, fluorescence intensities of 6 spots were increased and 12 were decreased in the dams. Representative 2-D DIGE gel images with differentially expressed POA spots are shown in Fig. 1. Fold changes of fluorescence intensities between spots of samples collected from mother and pup-deprived rats were in the range of − 1.35 to 1.66. The highest protein level increase in the POA region in dams was + 1.66 for Cryab. The highest protein level decrease in dams was triosephosphate isomerase (Tpi1, − 1.35).

### The Functional Clusters of the Maternally Altered Proteins in the POA Region

**Fig. 2:**
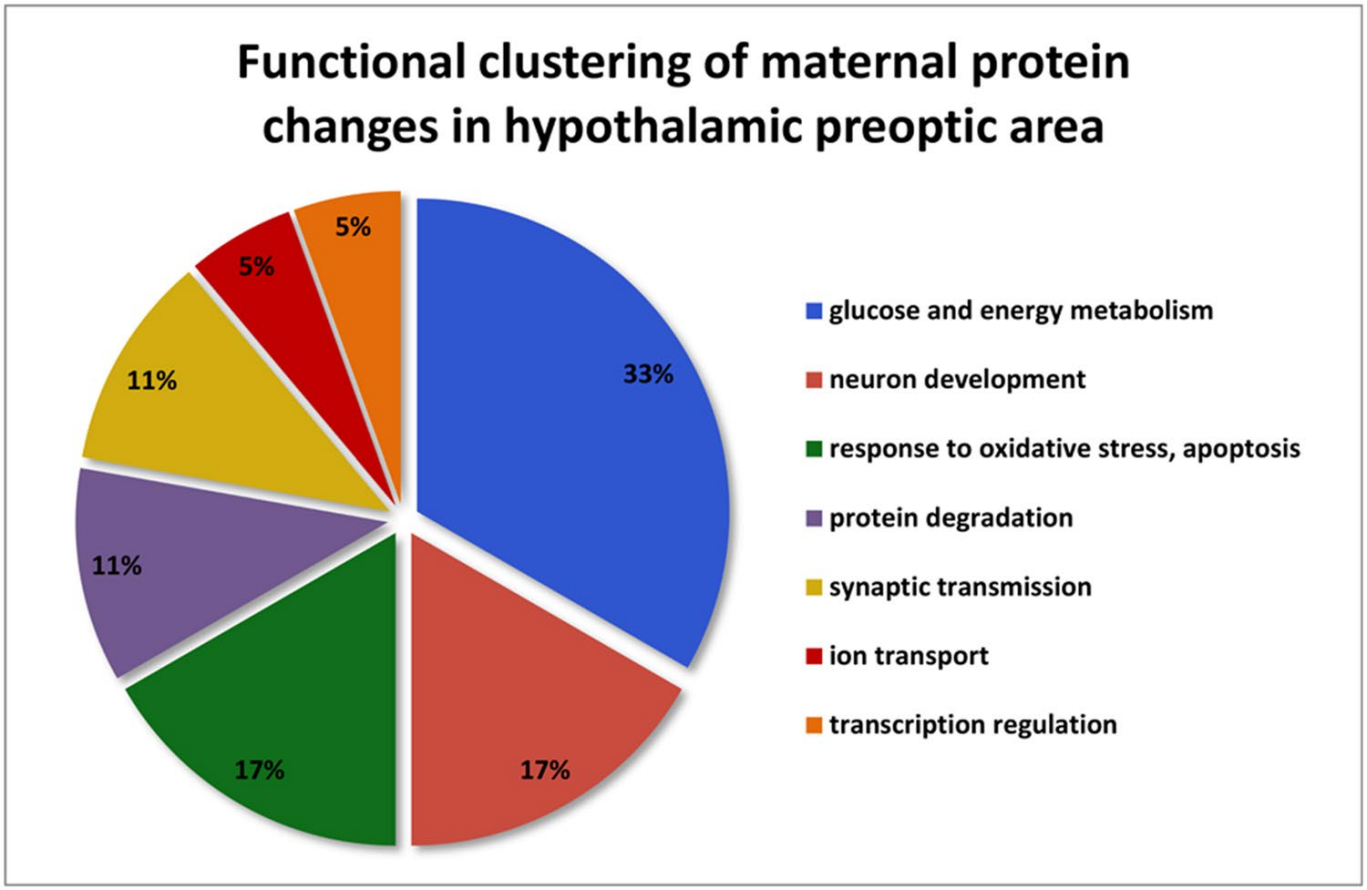
Functional clustering of maternal protein changes in hypothalamic preoptic areas. The altered and successfully identified 18 different proteins were analyzed. The major established functions of the 18 proteins were derived from the UniProt (http://www.UniProt.org/) and Gene Ontology (http://geneontology.org/) databases. The altered proteins participate in glucose and energy metabolism (n = 6), neuron development (n = 3), response to oxidative stress and apoptosis (n = 3), protein degradation (n = 2), synaptic transmission (n = 2), ion transport (n = 1) and transcription regulation (n = 1)

The altered proteins participate in glucose and energy metabolism (n = 6), neuron development (n = 3), response to oxidative stress and apoptosis (n = 3), protein degradation (n = 2), synaptic transmission (n = 2), ion transport (n = 1) and transcription regulation (n = 1). Thus, glucose and energy metabolism is the process to which most of the altered proteins in the POA belong (Fig. 2).

### Validation of Increased Level of Cryab

**Fig. 3:**
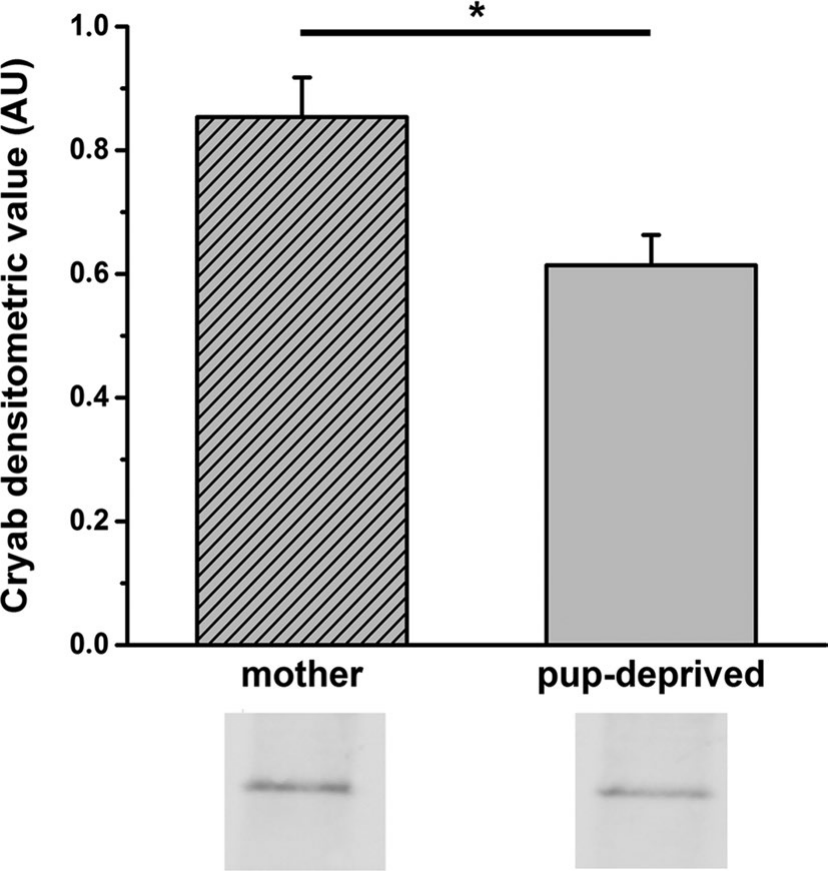
Western blot validation of changes in Cryab protein expression in the hypothalamic preoptic area of mother and pup-deprived female rats. Normalized densitometric values and representative immunopositive bands of Cryab are shown in the figure (n = 6, Student’s *t*-test, *p < 0.05; mean ± s.e.m. is shown)

WB analysis was performed for Cryab in the POA region (Fig. 3). The protein levels of Cryab (1.39 ± 0.15) were significantly increased (p < 0.05) in dams compared to pup-deprived rats. Thus, the WB results of the Cryab protein confirmed the 2-D DIGE data where the protein showed a 1.66-fold increase in its level in mothers.

### Common Regulators and Targets of Maternally Altered Proteins

**Fig. 4:**
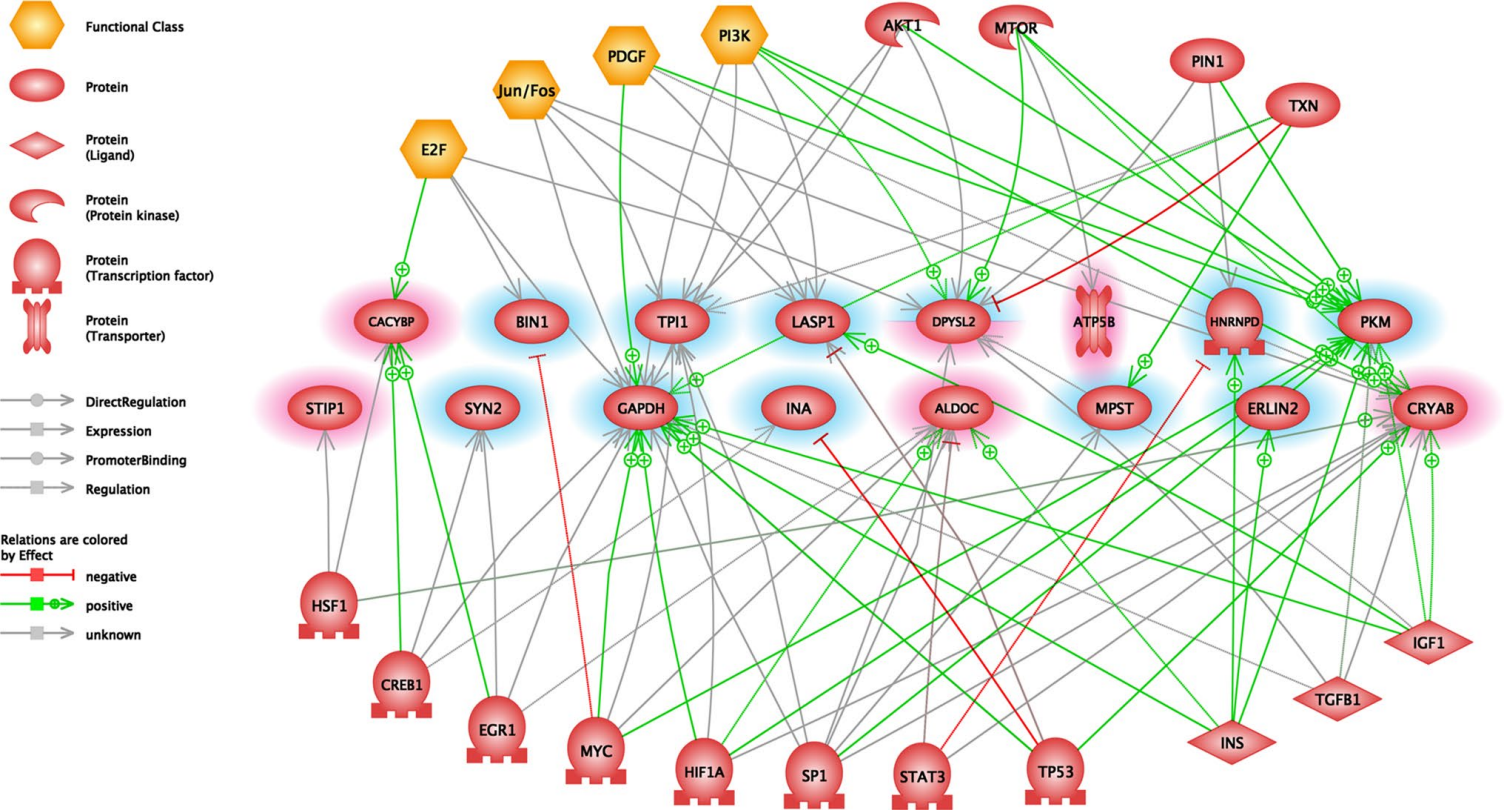
Common regulator analysis of hypothalamic preoptic areal proteins. *Abbreviations*: Transcription factor E2f (E2f), Transcription factor AP-1/Proto-oncogene c-Fos (Jun/Fos), Platelet-derived growth factor (Pdgf), Phosphatidylinositol-3-kinases (Pi3k), Rac-alpha serine/threonine-protein kinase (Akt1), Serine/threonine-protein kinase mTOR (Mtor), Peptidyl-prolyl cis–trans isomerase NIMA-interacting 1 (Pin1), Thioredoxin (Txn), Heat shock factor protein 1 (Hsf1), Cyclic AMP-responsive element-binding protein 1 (Creb1), Early growth response protein 1 (Egr1), Myc proto-oncogene protein (Myc), Hypoxia-inducible factor 1-alpha (Hif1a), Transcription factor Sp1 (Sp1), Signal transducer and activator of transcription 3 (Stat3), Cellular tumor antigen p53 (Tp53), Insulin (Ins), Transforming growth factor beta-1 (Tgfb1). Edges indicate the relationships between common regulators and altered maternal proteins. Red or blue highlights indicate maternal proteins that were significantly increased or decreased. Full protein names are presented in Fig. 6. (Color figure online)

With the Elsevier Pathway Studio Platform software, we could obtain comprehensive information on the proteins that interact with proteins altered in our study. This analysis extends our results although our technique can detect only a small proportion of the actually changing proteins. We identified the following proteins as regulators of several of the maternally altered proteins (Fig. 4) using common regulator analysis: Transcription factor E2f (E2f), Transcription factor AP-1/Proto-oncogene c-Fos (Jun/Fos), Platelet-derived growth factor (Pdgf), Phosphatidylinositol-3-kinases (Pi3k), Rac-alpha serine/threonine-protein kinase (Akt1), Serine/threonine-protein kinase mTOR (Mtor), Peptidyl-prolyl cis–trans isomerase NIMA-interacting 1 (Pin1), Thioredoxin (Txn), Heat shock factor protein 1 (Hsf1), Cyclic AMP-responsive element-binding protein 1 (Creb1), Early growth response protein 1 (Egr1), Myc proto-oncogene protein (Myc), Hypoxia-inducible factor 1-alpha (Hif1A), Transcription factor Sp1 (Sp1), Signal transducer and activator of transcription 3 (Stat3), Cellular tumor antigen p53 (Tp53), Insulin (Ins), and Transforming growth factor beta-1 (Tgfb1).

**Fig. 5:**
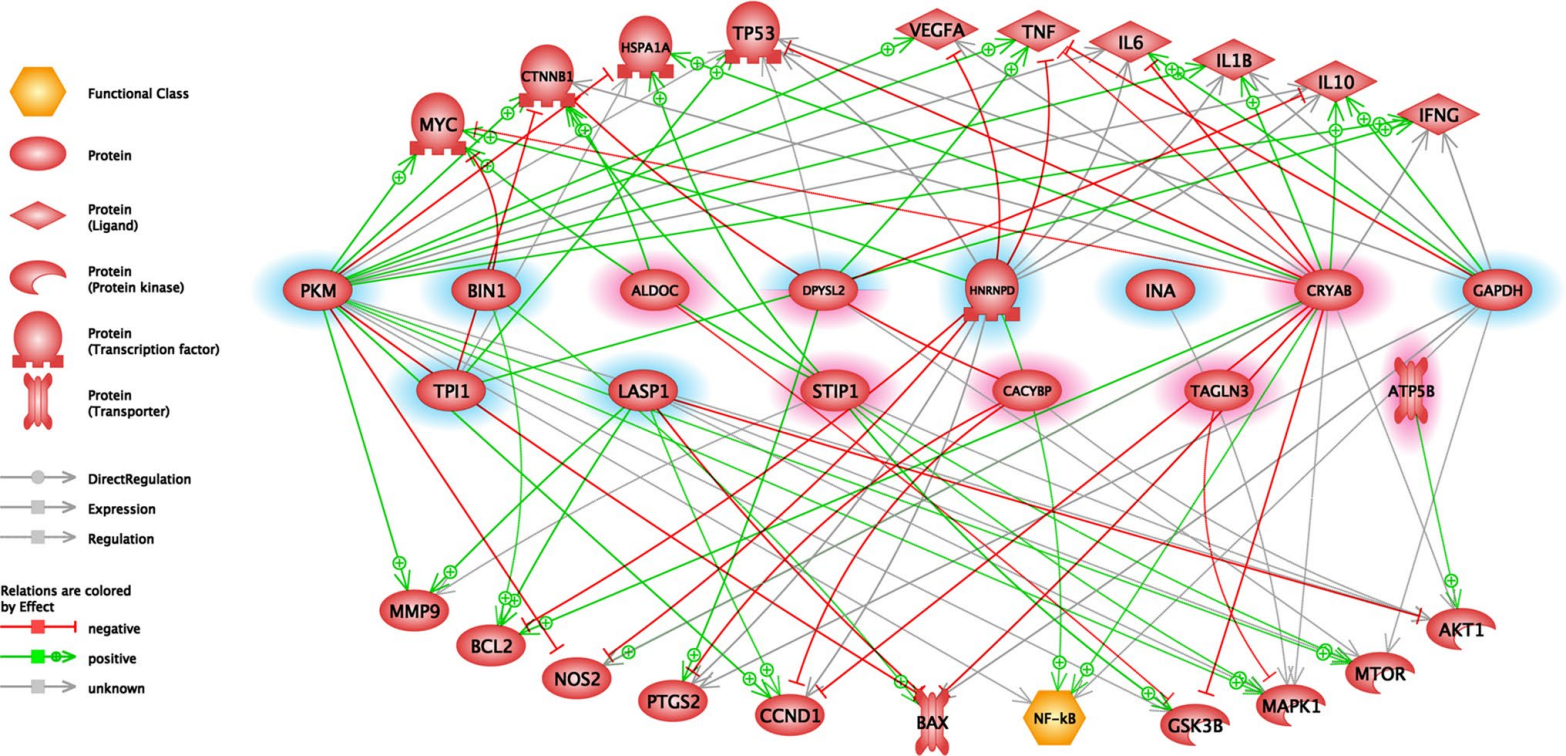
Common target analysis of hypothalamic preoptic areal proteins. Catenin beta-1 (Ctnnb1), Heat shock 70 kDa protein 1A (Hspa1a), Vascular endothelial growth factor A (Vegfa), Tumor necrosis factor (Tnf), Interleukin-6 (Il6), Interleukin-1 beta (Il1b), Interleukin-10 (Il10), Interferon gamma (Ifng), Matrix metalloproteinase-9 (Mmp9), Apoptosis regulator Bcl-2 (Bcl2), Nitric oxide synthase, inducible (Nos2), Prostaglandin G/H synthase 2 (Ptgs2), G1/S-specific cyclin-D1 (Ccnd1), Apoptosis regulator Bax (Bax), Nuclear factor NF-kappa-B (Nf-kb), Glycogen synthase kinase-3 beta (Gsk3b), Mitogen-activated protein kinase 1 (Mapk1). Edges indicate the relationships between common regulators and altered maternal proteins. Red or blue highlights indicate maternal proteins that were significantly increased or decreased. Full protein names are presented in Fig. 6. (Color figure online)

The following maternally altered proteins also have common targets according to our analysis of literature based databases (Fig. 5): Myc, Catenin beta-1 (Ctnnb1), Heat shock 70 kDa protein 1A (Hspa1a), TP53, Vascular endothelial growth factor A (Vegfa), Tumor necrosis factor (Tnf), Interleukin-6 (Il6), Interleukin-1 beta (Il1b), Interleukin-10 (Il10), Interferon gamma (Ifng), Matrix metalloproteinase-9 (Mmp9), Apoptosis regulator Bcl-2 (Bcl2), Nitric oxide synthase, inducible (Nos2), Prostaglandin G/H synthase 2 (Ptgs2), G1/S-specific cyclin-D1 (Ccnd1), Apoptosis regulator Bax (Bax), Nuclear factor NF-kappa-B (Nf-kb), Glycogen synthase kinase-3 beta (Gsk3b), Mitogen-activated protein kinase 1 (Mapk1), Mtor, Akt1.

**Fig. 6:**
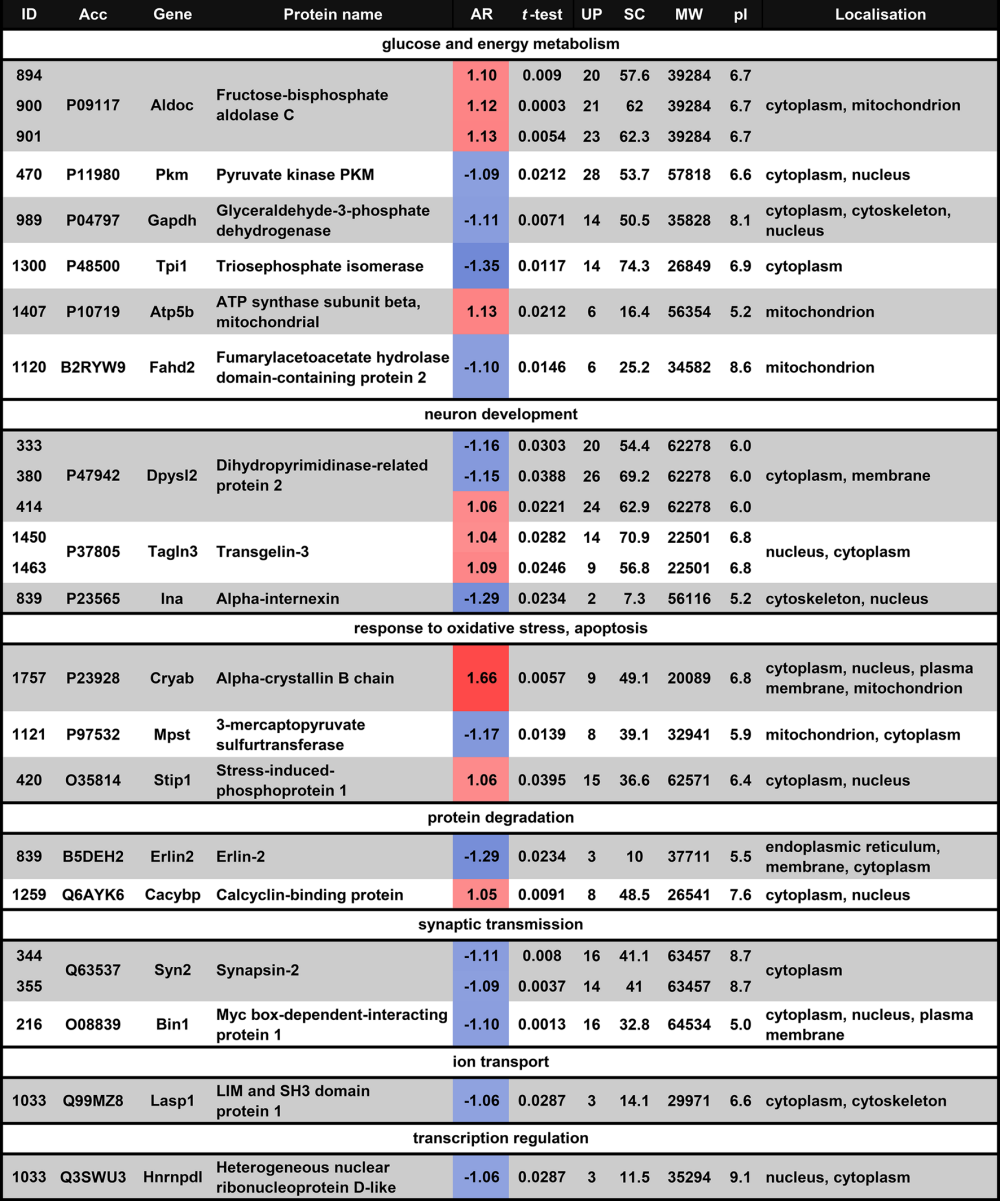
Significantly altered proteins in the hypothalamic preoptic area of mother rats assigned to functional clusters. The color gradient from red (elevated protein level) to blue (reduced protein level) is used to show the level of differential abundances of maternal proteins (the numbers represent the average ratio values). *Acc* accession number, *AR* average ratio, *UP* unique peptides number, *SC*% sequence coverage percentage, *MW* molecular weight, *pI* isoelectric point. (Color figure online)

## Discussion

We present here the first maternal proteomic study of the POA region. We identified a number of proteins with maternally altered levels, even though the 2-D DIGE technique we used does not allow for the investigation of all proteins since hydrophobic membrane proteins, proteins with a high molecular weight or with an extreme isoelectric point are not detected [37, 38]. Another limitation of the technique is that one spot can include multiple proteins due to insufficient protein separation efficiency, and we considered only the most abundant proteins in each spot in further analyses.

### Potential Functions of the Altered Proteins

There were a number of proteins altered in rat dams in the POA. The relationship between mothers and children is the strongest social bond, in which the POA plays a fundamental role [39, 40]. The transition to a maternal brain requires an adaptation in plasticity [41–43] and a great change in metabolism, which is reflected in the large number of cells with increased neuronal activity in the maternal POA [44, 45]. In this study, most of the altered proteins belong to glucose and energy metabolism and response to oxidative stress, which are likely correlated with the increased metabolic demand required by the maternally active neurons. Other altered proteins are involved in protein degradation, neuron development, synaptic transmission, ion transport and transcription regulation. These genes may play a role in the neuroplastic events, which accompany the maternal adaptation of the POA neurons [46, 47].

### Comparison with Previous Systems Biological Approaches in Mothers

In comparison with our previous proteomic study identifying maternally altered proteins in the mPFC, we found 9 proteins (Syn2, Dpysl2, Ina, Tagln3, Cryab, Stip1, Aldoc, Lasp1, and Cacybp) that were also changed in the POA. The direction of the changes is the same for 8 proteins (Syn2, Dpysl2, Tagln3, Cryab, Stip1, Aldoc, Lasp1, and Cacybp) [9] suggesting that the pool of maternally altered proteins overlap in different brain regions participating in maternal control. The reason behind this high degree of similarity could be that both brain regions had been exposed to the same hormonal environment. For example, female reproductive steroid hormones can regulate neuronal development and plasticity and energy metabolism in the mitochondria [48]. In addition, the changes that the neurons go through, such as neuroplasticity or a prolonged increase in neuronal activity, may also be similar.

Driessen et al. examined gene expression alterations in *postpartum* versus virgin mice to investigate potentially altered genes in maternal behavior [27]. Eleven of the altered proteins (Aldoc, Pkm2, Fahd2, Dpysl2, Cryab, Erlin2, Cacybp, Syn2, Stip1, Lasp1, and Hnrnpd) in our study showed the same direction of changes as their mRNA levels in the previous microarray study. In addition, four of the altered proteins (Aldoc, Ina, Cryab, and Stip1) were also changed at the mRNA level [49]. Although expressional changes at the mRNA and protein levels do not correlate very well with each other for all genes [50, 51], the similarities with previous mRNA results indicate that changes in the expression of some genes occur at both genomic and proteomic levels.

### Alpha-Crystallin B Chain (Cryab) and Its Potential Involvement in Maternal Adaptations

Cryab showed the highest degree of increase among the protein level changes in mother rats. In addition, Cryab has also been identified as a highly important maternally relevant gene based on its changes at the level of its mRNA expression [49]. Therefore, we further analyzed its expression level using an independent method and validated its change at the protein level with WB in a new set of samples. The successful confirmation of Cryab argues that the proteomics were well performed in our study. In addition, we also discussed the potential function of Cryab in central maternal adaptational processes.

Cryab is the major protein component of the vertebrate lens. However, its expression is not restricted to lenticular tissues; it has also been found in the heart, skeletal muscle and brain [52]. Overexpression of this protein has been found in several neurological disorders and pathological conditions, where Cryab can act as a neuroprotective protein [53]. Cryab belongs to the family of small heat shock proteins (Hsp20) and works as a molecular chaperone that holds proteins in large soluble aggregate forms [54]. It is presumed that its expression shows a correlation with cells with an elevated oxidative function [52]. By increasing its actions, the phosphorylated form of Cryab is involved in neuroprotection, cytoprotection after injury, responses to various types of stress and maintenance of the cytoskeleton [55–57]. Based on the previously established functions of Cryab, we suggest that the increased level of Cryab in the maternal POA contributes to the maintenance of functioning of neurons despite their elevated activity.

### Common Regulators and Targets of the Maternally Altered Proteins Suggest a Role of Prolactin in the regulation of Gene Expressional Changes

In the *postpartum* period, substantial changes can be observed in the hormonal level, and receptors of some maternally relevant hormones are abundant in the POA. The levels of steroid hormones (estrogen, progesterone) are very high at the end of pregnancy and drop robustly during lactation [58]. In contrast, the level of prolactin is high after delivery and remains high in the *postpartum* period as it is stimulated by suckling [19, 59–61]. Furthermore, the receptor level of prolactin is also known to be upregulated in the *postpartum* period [62]. The main signaling pathways activated by prolactin are the Jak/Stat, the Mapk/Erk, the Pi3k-Akt and the Rac [63]. Compared to the results of the common regulator and target analyses, many of the common regulators (Jun/Fos, Akt1, Mtor, Myc, and Stat3) and targets (Myc, Akt1, Mtor, Mapk1, Gsk3b, and Nos2) are also represented in the prolactin signaling pathway, suggesting that many of the protein level alterations arise from the action of prolactin via its receptor signaling pathway. However, additional pathways, e.g., those resulting from increased neuronal activity as a result of input from the pups, cannot be excluded either, as only a portion of the activated neurons in the mPOA respond to prolactin [5, 64].

## Conclusion

These results are the first to indicate that protein level changes take place in the POA as it adapts to the maternal challenges facing female rats in the *postpartum* period. Many of the changes correspond to previously identified alterations at the genomic level, while others were found only at the proteome level. Since the POA is known to control maternal behaviors, the identified proteins likely contribute to the behavioral changes of mother rats. Thus, they represent a molecular background of maternal behaviors. The previously established functions of the altered proteins suggest that most of them are involved in glucose metabolism, probably to elevate energy supply for the increased neuronal activity in the maternal POA. Proteins involved in neuroplasticity are also changed in mother rats, which is in line with the morphological changes taking place in the postpartum period. Increases in the level of Cryab were also individually validated. As a small heat shock protein, it may be involved in neuroprotection during the period of increased metabolic demand.

Since 10–15% of women suffer from *postpartum* depression after delivery, a much higher ratio than that of the general population, maladaptation to motherhood at the molecular level has been suggested to play a role in the development of depression [65, 66]. Thus, understanding the molecular events in the brain during adaptation to motherhood may also pave the way to establish mechanisms of maladaptation leading to *postpartum* depression.
